# Complete mitochondrial genomes of patients from Thailand with cardiovascular diseases

**DOI:** 10.1371/journal.pone.0307036

**Published:** 2024-07-11

**Authors:** Wipada Woravatin, Rattanasak Wongkomonched, Wichittra Tassaneeyakul, Mark Stoneking, Pattarapong Makarawate, Wibhu Kutanan

**Affiliations:** 1 Department of Biology, Faculty of Science, Khon Kaen University, Khon Kaen, Thailand; 2 Department of Biology, Faculty of Science, Naresuan University, Phitsanulok, Thailand; 3 Department of Pharmacology, Faculty of Medicine, Khon Kaen University, Khon Kaen, Thailand; 4 Department of Evolutionary Genetics, Max Planck Institute for Evolutionary Anthropology, Leipzig, Germany; 5 Biométrie et Biologie Évolutive, UMR 5558, CNRS & Université de Lyon, Lyon, France; 6 Department of Medicine, Faculty of Medicine, Khon Kaen University, Khon Kaen, Thailand; University of Campania, ITALY

## Abstract

Several previous studies have reported that both variation and haplogroups of mitochondrial (mt) DNA were associated with various kinds of diseases, including cardiovascular diseases, in different populations, but such studies have not been carried out in Thailand. Here, we sequenced complete mtDNA genomes from 82 patients diagnosed with three types of cardiovascular disease, i.e., Hypertrophic Cardiomyopathy (HCM) (*n* = 26), Long Q-T Syndrome (LQTS) (*n* = 7) and Brugada Syndrome (BrS) (*n* = 49) and compared these with 750 previously published mitogenome sequences from interviewed normal individuals as a control group. Both patient and control groups are from the same geographic region of northeastern Thailand. We found 9, 2, and 5 novel mutations that were not both damaging and deleterious in HCM, LQTS, and BrS patients, respectively. Haplogroup R9c was significantly associated with HCM (*P* = 0.0032; OR = 62.42; 95%CI = 6.892–903.4) while haplogroup M12b was significantly associated with LQTS (*P* = 0.0039; OR = 32.93; 95% CI = 5.784–199.6). None of the haplogroups was found to be significantly associated with BrS. A significantly higher density of mtDNA variants in the rRNA genes was found in patients with HCM and BrS (*P* < 0.001) than in those with LQTS or the control group. Effects of detected SNPs in either protein coding or tRNA genes of all the mitogenome sequences were also predicted. Interestingly, three SNPs in two tRNA genes (*MT-TA* m.5618T>C and m.5631G>A heteroplasmic variants in two BrS patients and *MT-TQ* m.4392C>T novel homoplasmic variant in a HCM patient) were predicted to alter tRNA secondary structure, possibly leading to abnormal tRNA function.

## Introduction

Cardiovascular diseases are diseases of the heart and blood vessels, e.g., cardiomyopathy and atrial fibrillation [1]. Over the past decade, the mortality rate attributed to cardiovascular diseases in the Thai population has been increasing, accounting for 23% of all deaths in 2016 and being a major cause of death in Thailand [2]. Some subtypes of cardiovascular disease, e.g., hypertrophic cardiomyopathy (HCM), long Q-T syndrome (LQTS), and Brugada syndrome (BrS) could lead to heart failure or sudden cardiac death in young adults aged less than 35 years old [3–5]. Based on functional and pathological criteria, HCM is a type of cardiomyopathy that represents a clinically and genetically heterogeneous group of cardiac disorders primarily involving the myocardium [6, 7]. While the incidence of these diseases in Thailand has not been individually reported, the estimated incidence of HCM in the general population was about 0.2% [8]; meanwhile, the estimated incidence of BrS in Asian and Southeast Asian countries is 0.15% and 0.27%, respectively [9]. The prevalence of LQTS has not been reported worldwide but it has a relatively low prevalence (approximate 0.04%) in Italy [4]. Multiple autosomal genetic loci have been found to be associated with these diseases, e.g., mutations in genes encoding sarcomere proteins in HCM patients [10] and genes encoding sodium ion channel proteins in LQTS patients [11, 12]. However, the genetic etiology of the diseases in some cases remains unknown.

Mitochondria are cellular organelles that have an essential function in the production of energy substrates (adenosine triphosphate, ATP) for mammalian cells, and play an important role in cell signaling pathways and programmed cell death [13]. Mitochondria are the major source of reactive oxygen species that are implicated in various cardiovascular diseases [14]. The mitochondria have their own genome, i.e., mitochondrial (mt) DNA, a double-stranded circular DNA of ~16,569 base pairs. The mtDNA encodes 37 genes, of which 13 are protein-coding genes for cellular respiration, oxidative phosphorylation, and ATP production [13, 15]. Missense mutations in these mtDNA genes could lead to abnormal cellular function and thus play a role in the development of cardiovascular diseases [16, 17]. Several mtDNA polymorphisms have been reported to be associated with some types of heart disease in different populations, e.g., *MT-ATP6* m.9104C>G and *MT-ND4L* m.10530G>A in patients diagnosed with LQTS from an Iranian population [18], and *MT-RNR2* m.2336T>C in Han-Chinese HCM patients [19]. In addition, many human diseases were associated with point mutations in mitochondrial tRNA genes [13]. Five genes, including *MT-TL*, *MT-TK*, *MT-TM*, *MT-TQ* and *MT-TI*, that encode tRNA-Leu, tRNA-Lys, tRNA-Met, tRNA-Gln and tRNA-Ile were identified as hot spots of mtDNA mutations for cardiovascular diseases [20] and over half of disease-associated mtDNA mutations were identified in genes encoding tRNAs [13]. However, some cardiovascular disease-associated mutations were also found with lower prevalence in other mtDNA regions, including the non-coding control region and genes encoding 12S and 16S rRNA (*MT-RNR1* and *MT-RNR2*, respectively) [19, 21–23].

The mtDNA mutations that have accumulated during the evolution and migration of modern humans can be used to assign haplogroups: a collection of haplotypes (sequences) characterized by specific alleles at a particular position in the mitochondrial genome [24]. Although haplogroup-associated mtDNA variations are not pathogenic, they may not be completely neutral; haplogroup-associated mtDNA variations could affect the assembly of the respiratory chain components [25] and the efficiency of the electron transport chain [26]. They have also been reported as either risk or protective factors for the development of ischemic cardiomyopathy [14]. Moreover, different mtDNA haplogroups may contribute to complex diseases in various populations [27, 28]. For example, haplogroup HV was associated with HCM in a Danish population [29], while haplogroup M10 was associated with HCM in Chinese families [30].

In this study, we sequenced complete mitochondrial genomes of HCM (*n* = 26), LQTS (*n* = 7) and BrS (*n* = 49) patients from northeastern Thailand. We identified mtDNA variants and haplogroups and then tested the differences in distribution of the haplogroups, variant frequencies, and variant density in each region between patient and control groups. We also characterized pathology-related mutations within the tRNA genes and predicted the impact on tRNA secondary structure *in silico*, based on the altered tRNA sequences.

## Materials and methods

### Samples

Ethical approval for this study was provided by the Khon Kaen University Ethics Committee in Human Research (Protocol No. HE611024). Since the number of patients diagnosed with hypertrophic cardiomyopathy (HCM), long QT syndrome (LQTS), and Brugada syndrome (BrS) by medical doctors at the Queen Sirikit Heart Center of the Northeast and Srinagarind Hospital, Faculty of Medicine, Khon Kaen University, was higher than for other types of cardiovascular disease, we selected these three groups for our study. Samples were recruited during April to August 2019. Written informed consent was obtained prior to collecting blood samples from all 82 participants (26 HCM patients, 7 LQTS patients, and 49 BrS patients). General information concerning the patients is provided in Table 1. The HCM participants were defined by a wall thickness ≥15 mm in one or more left ventricular myocardial segments as measured by any imaging technique, such as echocardiography, cardiac magnetic resonance imaging or computed tomography, that was not explained solely by loading conditions [31]. The criteria for LQTS recruitment were as follows: patients with either corrected QT interval (QTc) ≥480 ms in repeated 12-lead Electrocardiograms (ECGs) or LQTS risk score >3, or the presence of a QTc ≥460 ms in repeated 12-lead ECGs in patients with an unexplained syncopal episode in the absence of secondary causes for QT prolongation [32]. For the BrS cases, diagnostic criteria were ECGs with ST-segment elevation with type 1 morphology ≥2 mm in one or more leads among the right precordial leads V1 and/or V2 positioned in the second, third, or fourth intercostal space, occurring either spontaneously or after provocative drug test with intravenous administration of sodium channel blockers [32]. All sample donors were from northeastern Thailand. Genomic DNA was extracted using the QIAamp DNA Blood Mini Kit (QIAGEN, Hilden, Germany) according to the manufacturer’s protocol.

**Table 1:** General characteristics of patients diagnosed with three types of cardiovascular disease.

Characteristics	Hypertrophic cardiomyopathy (HCM)	Long Q-T syndrome (LQTS)	Brugada syndrome (BrS)
Number of samples	26	7	49
Age, Mean± Standard deviation, years	63±13	45±17	63±13
Age, Range, years	25–84	17–59	19–70
Male/female ratio	11/15	3/4	44/5
Family history of heart disease (the same type as proband)	5 (19)	0 (0)	19 (39)
Hypertension	4 (15)	1 (14)	7 (14)
Overweight/obesity	6 (23)	3 (43)	14 (29)
ICD* & Symptomatics	3 (12)	6 (86)	42 (85)

*ICD (Implantable Cardioverter Defibrillation) is a small-battery powered device placed in the patient chest to monitor heart rhythm or deliver electric shocks to fix an abnormal heart rhythm in patients with arrhythmia.

### DNA sequencing

During September 2019 to January 2020, complete mtDNA sequences were generated from genomic libraries prepared with double indices and enriched for mtDNA as described previously [33, 34]; the libraries were sequenced on an Illumina Hiseq 2500 to obtain mtDNA consensus sequences as described previously [35–37]. We used Bustard for Illumina standard base calling, and the read length was 76 bp. The procedures for manipulating raw sequencing data, alignment, and post-processing pipeline of the mtDNA sequencing data, including detection of mtDNA heteroplasmies, were carried out as previously described [35–37]. The mtDNA consensus sequences and mtDNA heteroplasmies were identified as described previously [38] using a Snakemake pipeline [39] (https://github.com/alexhbnr/StonekingLab_mtDNApipeline). We then manually checked and manipulated sequences with Bioedit [40] (www.mbio.ncsu.edu/BioEdit/bioedit.html). The complete mtDNA sequence data set has been deposited in GenBank (accession number PP273015-PP273096).

### Genetic and statistical analyses

Haplogroups were assigned by Haplogrep2 [41] with PhyloTree mtDNA tree Build 17 (www.phylotree.org) [42]. All mtDNA variants, including disease-associated SNPs, were identified using MitoMaster (www.mitomap.org); the SNPs which were not found in the MITOMAP database were noted as novel variants. To analyze the effect of missense mutations in protein coding regions, Polyphen-2 [43] (http://genetics.bwh.harvard.edu/pph2) and Provean [44] (http://provean.jcvi.org/index.php) were used to examine the predicted biological effects of amino acid substitutions on the structure and function of the proteins. All tRNA gene variants and missense mutations which were predicted as possibly pathogenic and deleterious variants were selected to compare their frequencies in patients compared to controls in order to identify potential disease-associated SNPs. All heteroplasmic or homoplasmic tRNA gene variants were analyzed for possible pathogenesis-association. The two-dimensional (2D) structure and identification of polymorphic and pathogenic positions of the tRNA gene variants were accessed using the Mamit-tRNA database [45] (http://mamit-trna.u-strasbg.fr) and MitoTIP [46] (mitochondrial tRNA informatics predictor, available via MITOMAP (www.mitomap.org). Homoplasmic tRNA variants that were found with significantly higher frequencies in cases than controls and heteroplasmic tRNA variants were selected for *in silico* tRNA secondary structure analysis. The tRNA RNAfold server [47] (http://rna.tbi.univie.ac.at//cgi-bin/RNAWebSuite/RNAfold.cgi) was used to predict tRNA secondary structure based on the sequence. For checking the conservation of the point mutations, tRNA sequences were compared with 10 species (*Pan troglodytes*, *Bos taurus*, *Canis lupus familiaris*, *Equus caballus*, *Mus musculus*, *Rattus norvegicus*, *Cyprinus carpio*, *Drosophila melanogaster*, *Gallus gallus*, and *Xenopus laevis*) using MitoMaster.

For the control group, 750 previously published mtDNA sequences from local northeastern Thai populations were retrieved [37, 48]. The criteria for sample collection in those previous studies were from healthy volunteers with ages ranging from 18 to 70 years old, and unrelated for at least two generations. MtDNA sequences from the patient and control groups were aligned with the revised Cambridge Reference Sequence (rCRS) [49] using MAFFT 7.271 [50] and manually checked with BioEdit [40].

Statistical analyses were performed using GraphPad Prism version 8.0.0 for Windows, GraphPad Prism Software (GraphPad Software, Inc. USA) and all differences were considered significant at *p* < 0.05. Bonferroni corrections were applied to adjust *p*-values for multiple comparisons. SNP and haplogroup frequencies in patient and control groups were compared using the Chi-square contingency test. Odds ratios (OR) and 95% confidence intervals (CI) were used to evaluate the relationship between mtDNA variants (SNPs and haplogroups) in cases and controls for each type of disease. The frequencies of homoplasmic variants in each region of the mitochondrial genome, and the number of predicted possibly deleterious mutations, were compared between the control group and patients diagnosed with each cardiovascular disease subtype using the Kruskal-Wallis test, performed by IBM SPSS Statistics for Windows, Version 28.0 (IBM Corp., Armonk, NY). The Mann-Whitney U test was also used to compare the density of mtDNA variants in the control region relative to the coding region.

## Results

### The identification and distribution of mtDNA mutations

The information on mtDNA sequences and haplogroup results for all 82 samples are shown in S1 Table. Among 26 HCM patients, we found a total of 360 variable positions in their mtDNA genomes (10 in tRNA genes, 46 in rRNA genes, 81 in non-coding regions, and 223 in protein-coding regions, of which 62 are missense mutations). Nine variants have not been reported in the MITOMAP database and were not found in the control group (Table 2); seven of these were detected to be heteroplasmic. Interestingly, except for *MT-ATP6* m.8577A>C (rs1603221597), eight variants, including two missense variants (*MT-CO2* m.7861T>A and *MT-CO3* m.9345C>A), two 12S RNA variants (*MT-RNR1* m.1151C>A and m.1263G>T), three 16S RNA variants (*MT-RNR2* m.2268G>A, m.2613T>C and m.2778T>A) and one non-coding 3-basepair deletion (m.ACA574del) homoplasmic variant, have not been reported in the Single Nucleotide Polymorphism database (dbSNP). The predicted impact of the two missense variants, *MT-CO2* m.7861T>A (p.Asp92Glu) and *MT-CO3* m.9345C>A (p.Leu47Met), on protein function were benign and neutral, based on Polyphen-2 and Provean, respectively. In addition, there are 24 non-missense variants in this study that have been associated with other diseases (S2 Table).

**Table 2:** Novel mutations in patients diagnosed with HCM, LQTS, and BrS, based on Mitomaster and dbSNP, and impact on protein function predicted by Polyphen-2 and Provean.

Disease	Mutation	Nature	dbSNP ID	Gene	Consequence	Amino acid change	Conservation	Predicted impacts on protein function		Patient ID	Haplogroup
								Polyphen-2	Provean		
HCM	**m.ACA574d**	**Homoplasmy**	**Not reported**	**D-loop**	**Non-coding variant**	**None**	**Not determined**	**ND**	**ND**	**HCM17**	**M17c**
	**m.1151C>A**	**Heteroplasmy**	**Not reported**	***MT-RNR1***	**12s RNA Variant**	**None**	**36%**	**ND**	**ND**	**HCM23**	**N21+195**
	**m.1263G>T**	**Heteroplasmy**	**Not reported**	***MT-RNR1***	**12s RNA Variant**	**None**	**91%**	**ND**	**ND**	**HCM11**	**M21b2**
										**HCM20**	**M7b1a1e1**
										**HCM23**	**N21+195**
	**m.2268G>A**	**Heteroplasmy**	**Not reported**	***MT-RNR2***	**16s RNA Variant**	**None**	**73%**	**ND**	**ND**	**HCM24**	**M22a**
	**m.2613T>C**	**Heteroplasmy**	**Not reported**	***MT-RNR2***	**16s RNA Variant**	**None**	**55%**	**ND**	**ND**	**HCM13**	**F1a3**
	**m.2778T>A**	**Heteroplasmy**	**Not reported**	***MT-RNR2***	**16s RNA Variant**	**None**	**18%**	**ND**	**ND**	**HCM24**	**M22a**
	**m.7861T>A**	**Heteroplasmy**	**Not reported**	***MT-CO2***	**Missense Variant**	**p.Asp92Glu**	**36%**	**Benign**	**Neutral**	**HCM08**	**R9c1b1**
	m.8577A>C	Heteroplasmy	rs1603221597	*MT-ATP6*	Synonymous Variant	p.Leu17 =	55%	None	None	HCM08	R9c1b1
	**m.9345C>A**	**Homoplasmy**	**Not reported**	***MT-CO3***	**Missense Variant**	**p.Leu47Met**	**80%**	**Benign**	**Neutral**	**HCM10**	**R9b2**
LQTS	m.8577A>C	Heteroplasmy	rs1603221597	*MT-ATP6*	Synonymous Variant	p.Leu17 =	55%	None	None	LQTS06	F1a1a1
	**m.14274A>C**	**Heteroplasmy**	**Not reported**	***MT-ND6***	**Missense Variant**	**p.Leu134Val**	**18%**	**Probably damaging**	**Neutral**	**LQTS03**	**C7**
BrS	**m.1263G>T**	**Heteroplasmy**	**Not reported**	***MT-RNR1***	**12s RNA Variant**	**None**	**91%**	**ND**	**ND**	**BrS24**	**M51a1b**
	**m.2691T>C**	**Homoplasmy**	**Not reported**	***MT-RNR2***	**16s RNA Variant**	**None**	**100%**	**ND**	**ND**	**BrS06**	**F1f**
	**m.7356G>C**	**Homoplasmy**	**Not reported**	***MT-CO1***	**Missense Variant**	**p.Val485Leu**	**60%**	**Benign**	**Neutral**	**BrS21**	**D5a**
	m.8577A>C	Heteroplasmy	rs1603221597	*MT-ATP6*	Synonymous Variant	p.Leu17 =	55%	None	None	BrS31	F3a1
	**m.13681A>C**	**Heteroplasmy**	**Not reported**	***MT-ND5***	**Missense Variant**	**p.Thr449Pro**	**27%**	**Probably damaging**	**Neutral**	**BrS42**	**B5a1b1**

The variants that were not reported previously in dbSNP are highlighted in boldface. HCM, hypertrophic cardiomyopathy; LQTS, long Q-T syndrome; BrS, Brugada syndrome; ND, not determined. D-loop is 1126 bp non-coding displacement loop of mitochondrial DNA (position 16024–576).

For LQTS, a total of 120 variable positions were found in 7 patients (2 in tRNA genes, 4 in rRNA genes, 29 in the non-coding region, and 85 in the protein-coding genes), with two heteroplasmic mutations that were not reported in the MITOMAP database and were not found in the control group (Table 2). A missense variant, *MT-ND6* m.14274A>C, has not been reported in dbSNP and had predicted impact on protein function as probably damaging and neutral, based on Polyphen-2 and Provean, respectively. There were 22 missense mutations (S3 Table), and 14 non-missense mutations were defined as disease-associated variants (S2 Table).

Among 49 patients diagnosed with BrS, there were 431 variable positions (22 in tRNA genes, 41 in rRNA genes, 107 in the non-coding region, and 260 in the protein-coding genes, of which 70 are missense mutations) (S2 Table). Five variants, including two homoplasmic and three heteroplasmic mutations, were not reported in the MITOMAP database nor in the control group of this study (Table 2), Excepting *MT-ATP6* m.8577A>C (rs1603221597), four variants, including one 12S RNA heteroplasmic variant (*MT-RNR1* m.1263G>T), one 16S RNA homoplasmic variant (*MT-RNR2* m.2691T>C), one missense homoplasmic variant (*MT-CO1* m.7356G>C) and one missense heteroplasmic variant (*MT-ND5* m.13681A>C), have not been reported in dbSNP. The predicted impact of *MT-ND5* m.13681A>C (p.Thr449Pro) on protein function was probably damaging and neutral, and for *MT-CO3* m.7356G>C (p.Val485Leu) benign and neutral, based on Polyphen-2 and Provean, respectively. A total of 57 variants were reported as disease-associated in MITOMAP, thirty-five of which are non-missense mutations (S2 and S3 Tables). Because almost all pathogenic mutations were heteroplasmic, we also provide information concerning all the heteroplasmic mtDNA mutations from all patients diagnosed with HCM, LQTS and BrS in this study (S4 Table).

**Fig 1:**
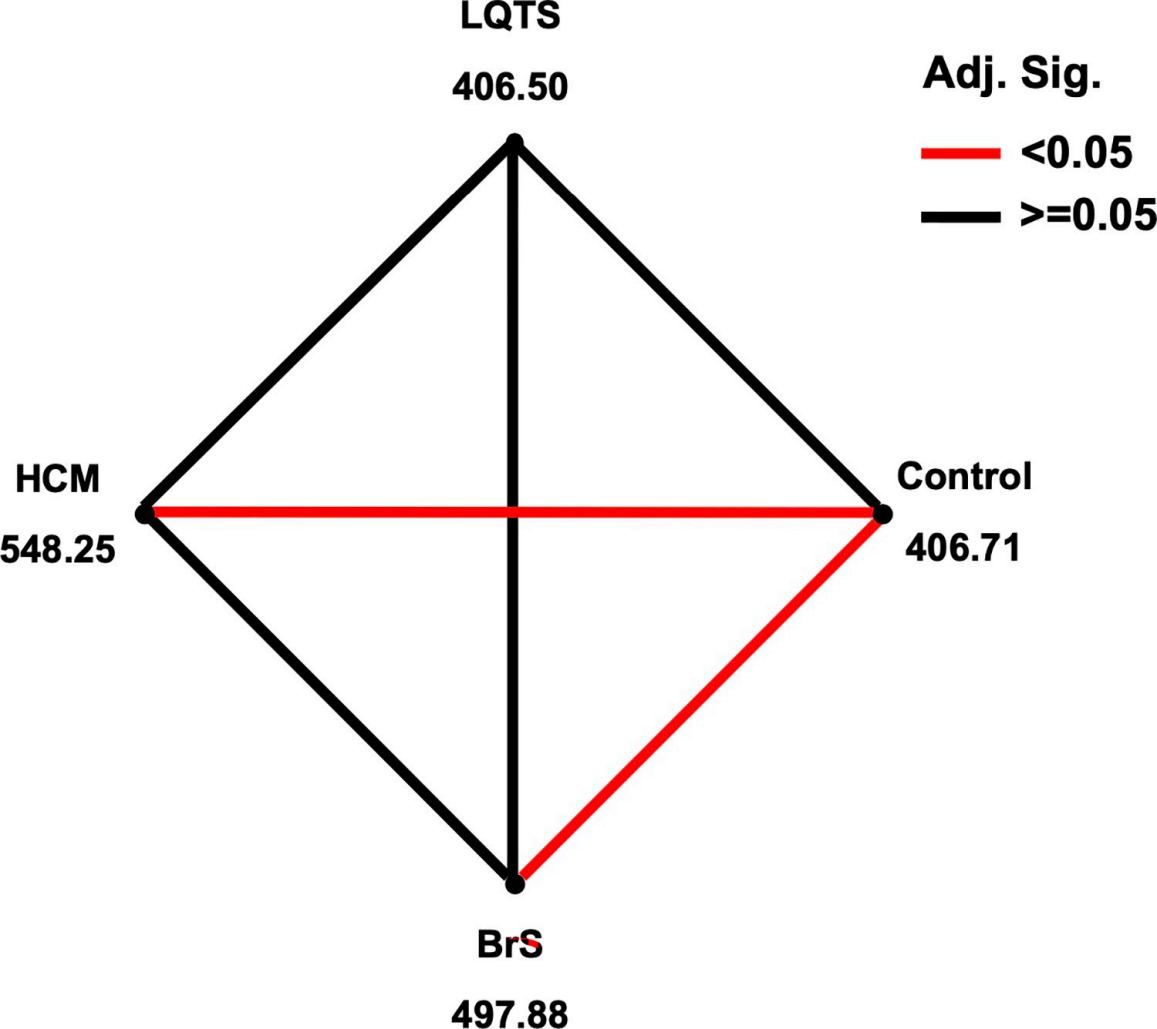
Comparative analyses of mtDNA variants among study groups (three patient groups and one control group). (A) The median of the total number of mtDNA variants per person. (B) Distribution of mtDNA variants in each region (Control region, tRNA, rRNA, and Protein coding region). (C) Pairwise comparison of mean rank between each pair of heart disease patient groups, and between each heart disease patient group and the control group. Numbers are the mean rank values for each group, and lines connect groups with significant differences (after Bonferroni adjustment) in the number of variants, as identified by the independent-samples Kruskal-Wallis test.

We compared the mtDNA mutation density of the whole genome and each region, including the control region, tRNA genes, rRNA genes, and protein-coding region, across the three patient groups and the control group. The median number of mtDNA variants per individual between the four studied groups is not significantly different (Fig 1A). All variants were then categorized into 4 mtDNA genomic regions for comparing each patient group with each other and with the control group. The overall results revealed a similar pattern of mtDNA variant distribution between HCM and BrS, which showed a higher proportion of rRNA variants than LQTS and controls (Fig 1B). Moreover, the pairwise comparison analysis also indicated that the rRNA variant frequency in both HCM and BrS was statistically significantly different from the control group. At the same time, other regions did not show a significant difference in variant numbers between any groups (Fig 1C). The number of variants in the control region was also compared to the coding region for all studied groups, including controls. All groups showed similar results, indicating a significantly higher variant frequency in the coding compared to the control region (*P* < 0.001).

### *In silico* pathogenic missense mutation analysis and the disease-associated SNPs

The results of *in silico* pathogenesis analysis, using the Polyphen-2 and Provean programs, show that none of the novel missense mutations are predicted to be damaging or deleterious (Table 2), whereas seven missense mutations that have been previously reported in HCM and BrS each were found to be possibly damaging/deleterious variants. However, after applying Bonferroni correction, there was no significant difference in frequencies of any missense mutations between case and control groups. Interestingly, one potentially deleterious variant, *MT-ND4* m.11087T>C, was identified in two individuals but was absent in controls. Another two missense mutations, *MT-ND1* m.4135T>C and *MT-CYB* m.15077G>A, were found as heteroplasmies in HCM patients and were also inferred to be possibly damaging and deleterious mutations (Table 3). Moreover, these three positions are highly conserved (>91%) among 10 different animal species, which could reflect the importance of this position for protein function (Table 3). In addition, there are four other missense mutations observed only in patient groups and previously associated with other diseases, including *MT-ATP8* m.8462T>C and *MT-ND4* m.11928A>G in BrS patients (Table 3) and *MT-ND1* m.3335T>C and *MT-ND5* m.12662A>G in HCM patients (Table 4). Both *MT-ATP8* m.8462T>C and *MT-ND4* m.11928A>G were predicted as deleterious mutations but *MT-ND1* m.3335T>C and *MT-ND5* m.12662A>G were not.

**Table 3:** Missense mutations in patients diagnosed with HCM, LQTS, and BrS that are predicted to be possibly damaging by Polyphen-2 and deleterious by Provean.

Disease	SNP	Nature	dbSNP ID	Gene	Amino acid change	Number of Cases	Number of Controls	P-value^a^	Conservation	Clinical Significance^b^
										ClinVar
HCM	m.4135T>C	Heteroplasmy	rs876661355	*MT-ND1*	p.Tyr277His	1	NA	NA	91%	Benign^c^
HCM	m.8531A>G	Homoplasmy	rs1556423481	*MT-ATP8*	p.Thr56Ala	1	2	0.0973	82%	Likely-Benign^c^
				*MT-ATP6*	p.Asn2Ser					
HCM	m.8603T>C	Homoplasmy	rs1603221627	*MT-ATP6*	p.Phe26Ser	1	1	0.0659	36%	Benign^c^
HCM	m.11087T>C	Homoplasmy	rs28433448	*MT-ND4*	p.Phe110Leu	2	0	0.0011	100%	Benign^c^
HCM	m.14180T>C	Homoplasmy	rs200933339	*MT-ND6*	p.Tyr165Cys	1	2	0.0973	18%	Benign^c^
HCM	m.15077G>A	Heteroplasmy	rs201943501	*MT-CYB*	p.Glu111Lys	1	NA	NA	91%	Benign^c^
HCM	m.15297T>C	Homoplasmy	rs1603225206	*MT-CYB*	p.Ile184Thr	1	1	0.0659	73%	Not Reported
BrS	m.3571C>T	Homoplasmy	rs200453691	*MT-ND1*	p.Leu89Phe	1	1	0.119	82%	Benign^c^
BrS	m.4824A>G	Homoplasmy	rs1556422903	*MT-ND2*	p.Thr119Ala	1	4	0.2719	64%	Benign^c^
BrS	m.8462T>C	Homoplasmy	rs1603221496	*MT-ATP8*	p.Tyr33His	1	0	0.0613	36%	Benign^c^
BrS	m.8572G>A	Homoplasmy	rs28502681	*MT-ATP6*	p.Gly16Ser	1	1	0.119	Not available	Benign^c^
				*MT-ATP8*	p.Ter69 =					
BrS	m.11928A>G	Homoplasmy	rs1569484466	*MT-ND4*	p.Asn390Ser	1	0	0.0613	64%	Benign^c^
BrS	m.12715A>G	Homoplasmy	rs1603223875	*MT-ND5*	p.Thr127Ala	1	8	0.4359	55%	Benign^c^
BrS	m.15077G>A	Homoplasmy	rs201943501	*MT-CYB*	p.Glu111Lys	1	1	0.119	91%	Benign^c^

HCM, hypertrophic cardiomyopathy; BrS, Brugada syndrome; NA, not available.

^a^Adjusted P-value after applying Bonferroni correction: HCM = 0.05/62 = 0.0008, LQTS = 0.05/22 = 0.0023, BrS = 0.05/70 = 0.0007.

^b^Specific standard terminology—"pathogenic", "likely pathogenic", "uncertain significance", "likely benign", and "benign" were used for describing variants identified in genes that cause Mendelian disorders [70].

^c^Leigh syndrome.

**Table 4:** Disease-associated SNPs observed only in patients diagnosed with HCM and BrS but not in controls.

Disease	SNP	Gene	Consequence	Amino acid change	dbSNP ID	Clinical Significance ^a^ (ClinVar)	Associated Diseases (Mitomaster)	References	No. of Cases	P-Value	OR	95% CI	Conservation
HCM	m.1192C>T	*MT-RNR1*	12S rRNA Variant	None	rs2068677739	Not reported	DEAF-associated	[50]	1	0.0335	Infinity	3.205 to Infinity	45%
HCM	m.3335T>C	*MT-ND1*	Missense Variant	p.Ile10Thr	rs879173824	Benign ^b^	LHON	[55]	1	0.0335	Infinity	3.205 to Infinity	73%
**HCM**	**m.4392C>T**	***MT-TQ***	**tRNA-Gln Variant**	**None**	**Not reported**	**Not reported**	**Poss. Hypertension factor**	**[18]**	**1**	**0.0335**	**Infinity**	**3.205 to Infinity**	**82%**
HCM	m.11365T>C	*MT-ND4*	Synonymous Variant	p.Ala202 =	rs28609979	Not reported	found in 1 HCM patient	[49]	1	0.0335	Infinity	3.205 to Infinity	100%
HCM	m.12662A>G	*MT-ND5*	Missense Variant	p.Asn109Ser	rs879105366	Benign ^b^	Recurrent pregnancy loss	[56]	1	0.0335	Infinity	3.205 to Infinity	9%
BrS	m.4659G>A	*MT-ND2*	Missense Variant	p.Ala64Thr	rs1556422882	Benign ^b^	LHON	[54, 57]	1	0.0613	Infinity	1.701 to Infinity	73%

One variant that was not reported previously in dbSNP is highlighted in boldface.

HCM, hypertrophic cardiomyopathy; BrS, Brugada Syndrome; OR, odds ratio; 95% CI, 95% confidence interval.

^a^ Specific standard terminology—"pathogenic", "likely pathogenic", "uncertain significance", "likely benign", and "benign" were used for describing variants identified in genes that cause Mendelian disorders [72].

^b^ Leigh syndrome.

**Fig 2:**
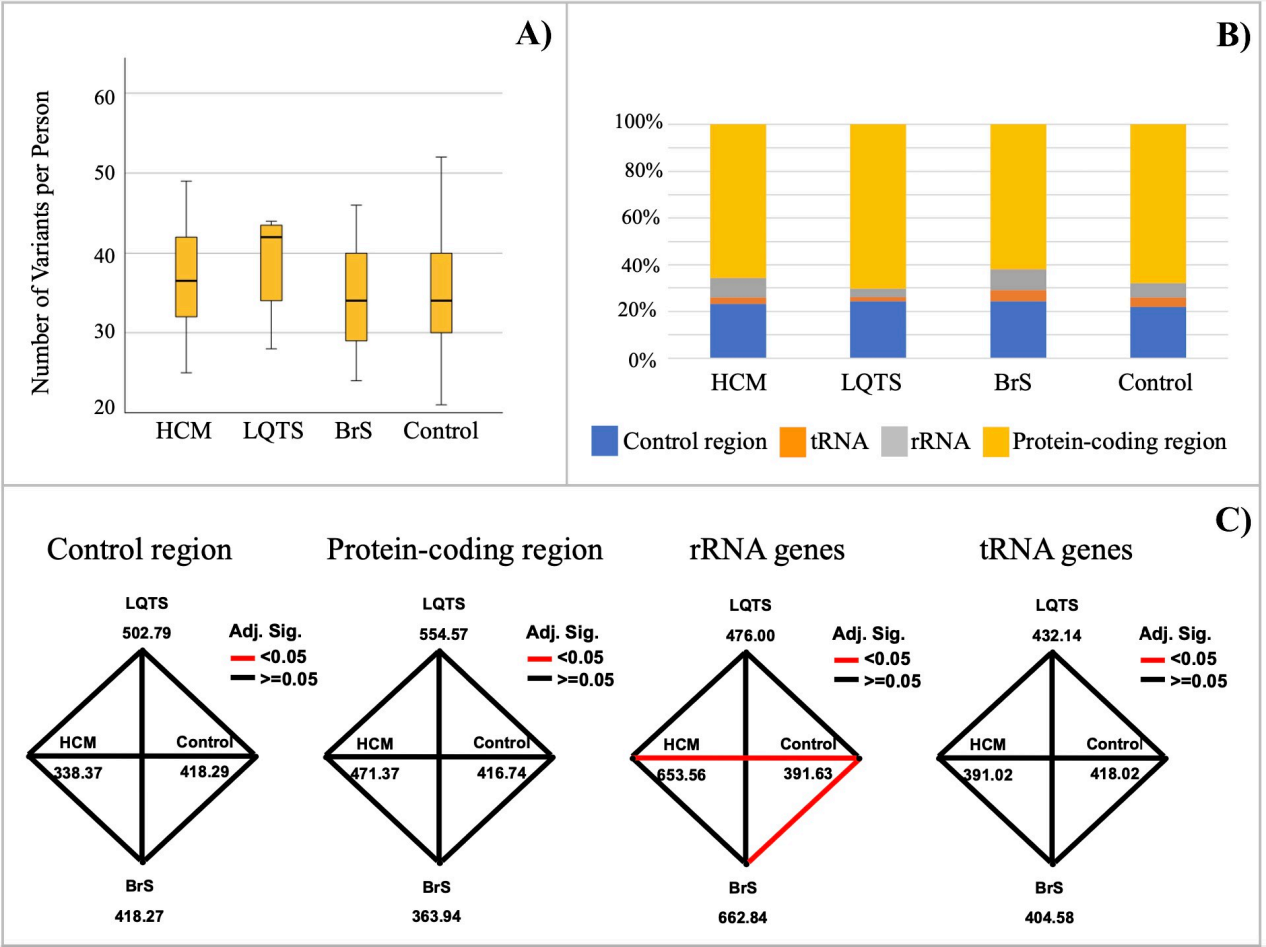
Pairwise comparison analysis of mean rank of predicted possibly damaging missense variants among study groups (three patient groups and one control group). Numbers are the mean rank values for each group, and lines connect groups with significant differences (after Bonferroni adjustment) in the number of variants, as identified by the independent-samples Kruskal-Wallis test.

Due to the lack of missense mutations which are strongly associated with cardiovascular disease in our patients, we did additional analysis by comparing the frequency of all possibly damaging missense mutations among the three patient groups and the control group. Based on the prediction results using Polyphen-2, there are 10, 1, 19, and 79 possibly deleterious homoplasmic missense variants found in the HCM, LQTS, BrS, and control groups, respectively (Table 3 and S5 Table). The frequencies of possibly damaging mutations in the two case groups, HCM and BrS, were significantly higher than in the control group (Fig 2) parallel with the greater number of rRNA gene variants observed in HCM and BrS patients (Fig 1). These higher mutation densities could possibly alter protein structure and function, playing a role in the development of these two diseases.

Among the disease-associated non-missense variants, we found *MT-RNR1* m.1192C>T, *MT-TQ* m.4392C>T, and *MT-ND4* m.11365T>C occurred in HCM patients but were absent in control subjects. However, after applying Bonferroni correction, the frequencies of all three mutations between case and control groups were not significantly different (S2 Table and Table 4). These SNPs were previously reported to be associated with other human diseases or mitochondrial dysfunction [20, 51, 52].

### mtDNA haplogroups of HCM, LQTS and BrS

HCM patients had 11 mtDNA haplogroups, i.e., B4a, B5a, F1a, F* (xF1), M7b, M* (xM7), N21+195, N9a, R* (xR9), R9c, and R9b. Only haplogroup R9c was significantly more frequent in the HCM patients (7.7%) than the controls (0.1%) (*p* = 0.0032; OR = 62.42; CI 95% = 6.892 to 903.4). Six mtDNA haplogroups, i.e., C7, F1a, F1f, M12b, M7b, and N21+195 were observed in LQTS patients and the prevalence of haplogroup M12b was significantly higher in the LQTS cases than the controls (28.6% vs 1.2%; *p* = 0.0039; OR = 32.93; CI 95% = 5.784–199.6). And the frequencies of all 14 mtDNA haplogroups found in the BrS patient group, i.e., B4a, B4b, B4c, B4g, B5a, C7, D4g, D5a, F1a, F1f, F3a, M* (x M7), M7b, N21+195, N9a, R* (xR9), and R9b were not significantly different from the control group.

### Identification of tRNA mutations and *in silico* tRNA secondary structure analysis

**Fig 3:**
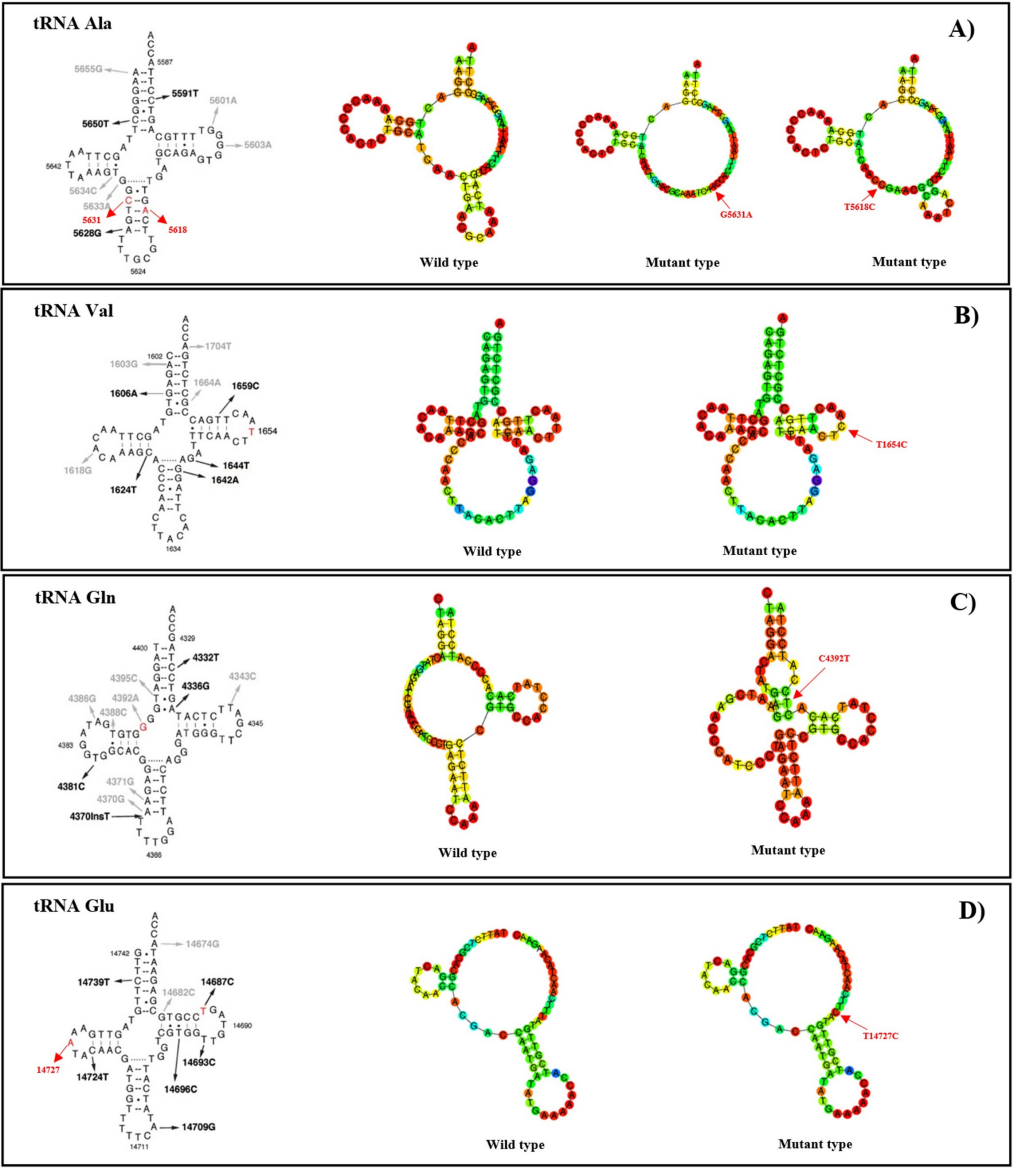
Location and predicted impact of gene variants on tRNA secondary structure. (A) *MT-TA* m.5631G>A and m.5618T>C alter the predicted secondary structure of tRNA-Ala. (B) *MT-TM* m.1654T>C does not alter the predicted secondary structure of tRNA-Val. (C) *MT-TQ* m.4392 C>T alters the predicted secondary structure of tRNA-Gln. (D) *MT-TE* m.14727T>C does not alter the predicted secondary structure of tRNA-Glu.

Over half of the disease-associated mtDNA mutations in previous studies are found in genes encoding tRNA, and many of these are heteroplasmic [20]. Therefore, we analyzed tRNA variants in more detail, especially the heteroplasmic variants (S6 Table). Three heteroplasmic tRNA gene mutations were found in patients diagnosed with HCM and BrS. The m.5618T>C variant was detected in a 50-year-old woman with asymptomatic BrS and no family history of BrS. Meanwhile, the m.5631G>A was observed in a 70-year-old man with symptomatic BrS and with a family history of BrS. Another heteroplasmic variant, *MT-TV* m.1654T>C, was detected in a 63-year-old man with symptomatic HCM but no family history of HCM. We further investigated the effects of these mutations on tRNA structure and predicted 2D structure using mammit-tRNA and RNAfold server. Both m.5618T>C and m.5631G>A variants are in the anticodon stem of tRNA-Ala and could cause instability in the secondary structure, compared with the wild type (Fig 3A). By contrast, *MT-TV* m.1654T>C in the T-loop is not predicted to affect the secondary structure (Fig 3B).

For homoplasmic tRNA mutations, none had a significantly higher frequency in HCM or BrS patients than in controls. However, *MT-TQ* m.4392C>T was found in a HCM patient and was not detected in the control group; this mutation was found in hypertension patients in a previous study [18], and the tRNA structure analysis indicates that m.4392C>T may alter the secondary structure of tRNA-Gln relative to the wild type (Fig 3C). One variant, *MT-TE* m.14727T>C located in the D-loop of tRNA-Glu, was significantly more frequent in the LQTS patients than controls. However, this variant is not predicted to alter the tRNA-Glu structure (Fig 3D).

## Discussion

Previous studies reported associations between mtDNA variants and various diseases, including cardiovascular disease, in different populations [14, 22, 23, 28]. In Thailand, the *MT-ND4* m.11778 G>A mutation was associated with Leber hereditary optic neuropathy (LHON) [53, 54] but mtDNA variation in cardiovascular disease patients has not been previously reported in Thailand. Here, we sequenced complete mtDNA genomes of patients with three cardiovascular disease subtypes, i.e., hypertrophic cardiomyopathy (HCM), long Q-T syndrome (LQTS) and Brugada syndrome (BrS). We found 9, 2, and 5 novel mutations, most of which were heteroplasmic, in patients diagnosed with HCM, LQTS, and BrS, respectively, although these variants are not predicted to be damaging or deleterious (Table 2). One heteroplasmic missense mutation, *MT-CYB* m.15077G>A, was predicted to be possibly damaging/deleterious; moreover, the ancestral allele is highly conserved among 10 different animal species (Table 3). The m.15077G>A mutation was previously associated with maternally inherited deafness (DEAF) and Leber’s Hereditary Optic Neuropathy (LHON) [55, 56], suggesting it could be potentially pathogenic for HCM. However, functional studies found that cybrids with m.15077G>A had normal enzymatic activities for all the respiratory chain complexes [55, 56].

In addition, five HCM-associated variants, including *MT-RNR1* m.1192C>T, *MT*-*ND1* m.3335T>C, *MT-TQ* m.4392C>T, *MT-ND4* m.11365T>C and *MT-ND5* m.12662A>G, and one BrS-related variant, *MT-ND2* m.4659T>C, were observed in the patients but not in controls (Table 4). The *MT-RNR1* m.1192C>T mutation was associated with nonsyndromic hearing loss (DEAF), based on the MitoMaster database. The m.1192C>T occurs at a highly conserved nucleotide within a loop of the 12S rRNA and alters tertiary or quaternary structure of the rRNA, leading to significant changes in rRNA function and subsequently contributing to the deafness phenotype [52]. This suggests that the m.1192C>T alteration could contribute to the HCM phenotype in this study, as reported in a previous study [52]. The *MT*-*ND1* m.3335T>C variant was reported in a Han Chinese patient with LHON and a functional study showed that the activity of complex I in the mutant cell lines containing this variant was only 70% relative to wild-type cell lines [57]. Therefore, m.3335T>C, which is a highly conserved position, and which results in a decrease in complex I activity, could be involved in HCM pathogenesis. The *MT-ND4* m.11365T>C variant was initially reported in a 65-year-old man with an uncommon form of HCM [51], suggesting this mutation may be related to HCM in this patient. The *MT-ND5* m.12662A>G variant was previously found in women with recurrent pregnancy loss (RPL) and not in healthy women [58]. Several mutations in the *MT-ND5* gene were found in RPL patients and suggested to be associated with pathogenesis of RPL. However, the m.12662A>G mutation always appeared with other RPL-associated mutations, not occurring alone in any patient; therefore, this mutation may not cause disease by itself [58]. *MT-ND2* m.4659G>A was previously associated with Parkinson’s disease (PD) and LHON patients in Russian Tatar and Western Siberian populations, respectively [56, 59]. This mutation was also previously reported to be associated with LHON in an Australian family [56], along with two heteroplasmic variants *MT-ND6* m.14484T>C and *MT-ND2* m.5460G>A. The *MT-ND2* m.4659G>A was found at a very low frequency in general populations, therefore this variant could possibly play a role in the development of BrS [56].

Several previous studies have reported a higher number of mutations in the protein coding regions than in the control region in patients with many diseases [60–62]. In contrast to those studies, we found a significantly higher density of variants in the coding region in both the three case groups and the controls. However, the frequencies of rRNA gene variants in HCM and BrS patients were significantly higher than in controls (Fig 1). Even though rRNA genes are not pathogenic mutation hotspots for cardiovascular disease, it is possible that the increase in number of both polymorphic and deleterious variants might affect the rRNA function and translation process in mitochondria, leading to mitochondrial dysfunction. For example, *MT-ATP6* m.8701A>G and *MT-ND3* m.10398A>G, even though these two variants were reported to be polymorphisms, might affect mitochondrial function and thus play a role in the etiology of complex diseases [63].

Overall, the patient and control groups had similar frequencies of mutations in the protein-coding region. Interestingly, possibly damaging mutations in HCM and BrS patient groups were significantly higher than in the control group (Fig 2). These missense mutations could possibly impact the function of the proteins involved in the electron transport chain, eventually leading to more severe symptoms of heart disease. However, these results should be interpreted with caution due to the unknown impact of these mutations on protein function.

Our mtDNA haplogroup results in the Thai population indicated that haplogroup R9c was significantly more frequent among HCM patients than the control group whereas haplogroup M12b appeared to be associated with LQTS. A non-coding variant, m.16129G>A, is one of the specific variants for identifying haplogroup M12b. However, a high frequency of the m.16129G>A variant was also found in controls (40.53%). There was no significant difference in frequency of the m.16129G>A between case and control groups. These might suggest that this variant alone may not induce pathogenesis, but together with other mutations it could contribute to development of the disease. In addition, heteroplasmy involving m.16129G>A was frequently detected in patients with migraine headache and cyclic vomiting syndrome [64]. In addition to m.16129G>A, *MT-ND5* m.12372G>A is a marker of haplogroup M12 and has been associated with Sporadic Creutzfeldt–Jakob disease (sCJD) in Chinese patients, and probably could change the pH in the brain, affecting the organ function [65]. The other marker variants for haplogroups R9c and M12b have not been reported as pathogenic mutations associated with any diseases. However, our case and control samples probably consist of several sub-populations, and so it is not clear if the association between haplogroup and disease is a true, causal association, or rather an artifact of population structure.

Previous studies reported associations between different tRNA mutations and many types of arrhythmogenic cardiomyopathy. Two *MT-TI* gene mutations, m.4300A>G and m.4277C>T, were associated with symptomatic HCM [66, 67]. The HCM patients who carry these two mutations had a large proportion of cytochrome c oxidase deficient cardiomyocytes and defects in activity for respiratory chain complexes I, III and IV. They also had decreased levels of tRNA-Ile expression [68]. In this study, we detect a heteroplasmic *MT-TV* gene mutation, m.1654T>C, in a 63-year-old man with symptomatic HCM and no family history of HCM. However, this variant is not inferred to affect tRNA-Val structure (Fig 3B). One homoplasmic *MT-TQ* gene variant, m.4392C>T, was detected in a HCM case but not in controls (S6 Table). This mutation could affect tRNA-Gln secondary structure (Fig 3C) and changes a highly conserved position (80% according to the MITOMAP database). The m.4392C>T mutation was previously reported in a hypertension patient from the Han-Chinese population and the inferred change in tRNA structure may cause translation impairment and affect protein synthesis [18]. In another previous study, two *MT-TQ* gene mutations, m.4377T>A and m.5580T>C, as well as two *MT-TM* gene mutations, m.4407G>A and m.4456C>T, were observed in BrS patients [69] but not in controls. Except for *MT-TQ* m.5580T>C, the other variants were inferred pathogenic mutations because of their heteroplasmic status and impact on the tRNA-Gln or tRNA-Met structure. This might suggest that primary pathogenic mutations tend to be heteroplasmic rather than homoplasmic variants. Here, two heteroplasmic mutations in the anticodon stem in *MT-TA* genes, m.5618T>C and m.5631G>A, from Thai patients diagnosed with BrS (S6 Table) could alter the secondary structure of tRNA-Ala (Fig 3A). The *MT-TA* m.5618T>C transition affects the highly conserved part of the acceptor arm of the tRNA-Ala structure, possibly leading to the abnormal function of the tRNAs. How such mutations could impact tRNA function is exemplified by a functional study of a mutation in the acceptor arm of tRNA-Thr, *MT-TT* m.15927G>A, that was associated with coronary heart disease in a Han-Chinese family [70]. The *MT-TT* m.15927G>A mutation changes a highly conserved nucleotide which is essential for tRNA stability and accurate aminoacylation. The cell lines carrying the m.15927G>A variant had an approximately 80 percent decrease in the steady-state level of tRNA-Thr and increased production of reactive oxygen species in the cells that could damage DNA, proteins, or membranes, leading to mitochondrial dysfunction and cell apoptosis [70]. This could be particularly important for tissues that are highly active metabolically, such as heart.

The second heterplasmic tRNA mutation found in this study, *MT-TA* m.5631G>A, was previously reported in a young patient with isolated myopathy and her mother [71]. The level of mtDNA heteroplasmy was higher in this patient’s muscle sample (92%), compared with the peripheral blood sample (77%). High mutation loads of the *MT-TA* m.5631G>A mutation in the patient’s cytochrome c oxidase (COX)-deficient fibers and decreased activities of respiratory chain complexes I, II, III, and IV confirms that this mutation is likely associated with the etiology of isolated myopathy in this case [71]. Thus, the *MT-TA* m.5631G>A mutation might also be pathogenic and associated with development of BrS. In addition, the frequency of a homoplasmic variant, *MT-TE* m.14727T>C, in Thai LQTS patients was significantly more frequent in cases than controls (S6 Table). However, this variant is not inferred to affect tRNA structure (Fig 3D) and has not been reported to be associated with any diseases.

In conclusion, we reported and analyzed complete mitochondrial genome sequences in 26 hypertrophic cardiomyopathy patients, 7 long Q-T Syndrome patients and 49 Brugada Syndrome patients from Northeastern Thailand. There were several limitations in this study that should be mentioned, such as the low sample sizes for the case groups, especially the long Q-T Syndrome group. Potential differences between case and control groups would be easier to detect with larger sample sizes. In addition, although the control subjects retrieved from previous population genetic studies were healthy and asymptomatic of any diseases at time of interview, additional tests, such as ECG recordings, from these control subjects could confirm that they were indeed free of heart disease. Moreover, we did not attempt to replicate these results in another population. Despite these limitations, we highlight the contributions of this study. First, we generated 82 complete mtDNA genomes of patients with three cardiovascular disease subtypes that were rarely studied previously in Thailand. Second, we highlighted the potential contribution of mtDNA variants to the development of these cardiovascular diseases. Third, both case and control groups have been recruited from the same geographic location, namely the Northeastern region of Thailand. Finally, we identified novel mutations and other SNPs that were disease-associated and affected tRNA secondary structure and are thus candidates for further study. Our study thus paves the way for further investigations of the role of mtDNA mutations in cardiovascular disease in Thailand. In addition, future genetic studies involving diverse populations can provide more comprehensive information about cardiovascular diseases.

## Supporting information

S1 Table MtDNA sequencing coverage and haplogroup of patients diagnosed with HCM, LQTS and BrS. (XLSX)

S2 Table Disease-associated SNPs observed in patients diagnosed with HCM, LQTS and BrS in relative to controls. (XLSX)

S3 Table Missense mutations detected in mitochondrial genomes of patients diagnosed with HCM, LQTS and BrS and impacts on protein function predicted by Prohen-2 and Provean. (XLSX)

S4 Table Heteroplasmic mitochondrial DNA mutations in patients diganosed with HCM, LQTS and BrS. (XLSX)

S5 Table Homoplasmic missense mutations in mitochondrial genome of controls and impacts on protein stucture predicted by Polyphen-2. (XLSX)

S6 Table Mutations in mitochondrially encoded tRNA genes of patients diagnosed with HCM, LQTS and BrS in relative to controls. (XLSX)
